# Synthesis and Biological Activity of Homohypotaurine Obtained by the Enzyme-Based Conversion of Homocysteine Sulfinic Acid Using Recombinant *Escherichia Coli* Glutamate Decarboxylase

**DOI:** 10.3390/molecules29173985

**Published:** 2024-08-23

**Authors:** Mario Fontana, Aysenur Gunaydin Akyildiz, Chiara D’Alonzo, Fabio Giovannercole, Arianna Zicchi, Antonio Francioso, Elisabetta Capuozzo, Daniela De Biase

**Affiliations:** 1 Department of Biochemical Sciences “A. Rossi Fanelli”, Sapienza University of Rome, Piazzale Aldo Moro 5, 00185 Roma, Italy; mario.fontana@uniroma1.it (M.F.); afrancioso@unite.it (A.F.); elisabetta.capuozzo@uniroma1.it (E.C.); 2 Department of Pharmaceutical Toxicology, Faculty of Pharmacy, Bezmialem Vakif University, 34093 Istanbul, Turkey; gunaydinaysenur@gmail.com; 3 Department of Medico-Surgical Sciences and Biotechnologies, Sapienza University of Rome, Corso della Repubblica 79, 04100 Latina, Italyfabio.giovannercole@gmail.com (F.G.); ariannazicchi@gmail.com (A.Z.)

**Keywords:** green chemistry, PLP, sulfinic compounds, HUVEC, H9c2, hydrogen peroxide

## Abstract

*l*-Homocysteine, formed from S-adenosyl methionine following demethylation and adenosine release, accumulates when the methionine recycling pathway and other pathways become impaired, thus leading to hyperhomocysteinemia, a biomarker in cardiovascular diseases, neurological/psychiatric disorders, and cancer. The partial oxidation of the *l*-homocysteine thiol group and its decarboxylation on C-alpha lead to the formation of *l*-homocysteinesulfinic acid (*l*-HCSA) and homohypotaurine (HHT), respectively. Both compounds are not readily available from commercial suppliers, which hinders the investigation of their biological activities. Herein, the chemical synthesis of *l*-HCSA, from *l*-homocystine, was the starting point for establishing the bio-based synthesis of HHT using recombinant *Escherichia coli* glutamate decarboxylase (*Ec*GadB), an enzyme already successfully employed for the bio-based synthesis of GABA and its phosphinic analog. Prior to HHT synthesis, *k*_cat_ (33.92 ± 1.07) and *K*_M_ (38.24 ± 3.45 mM) kinetic constants were determined for *l*-HCSA on *Ec*GadB. The results of our study show that the *Ec*GadB-mediated synthesis of HHT can be achieved with good yields (i.e., 40% following enzymatic synthesis and column chromatography). Purified HHT was tested in vitro on primary human umbilical vein endothelial cells and rat cardiomyoblasts and compared to the fully oxidized analog, homotaurine (OT, also known as tramiprosate), in widespread pharmaceutical use. The results show that both cell lines display statistically significant recovery from the cytotoxic effects induced by H_2_O_2_ in the presence of HHT.

## 1. Introduction

**Figure 1:**
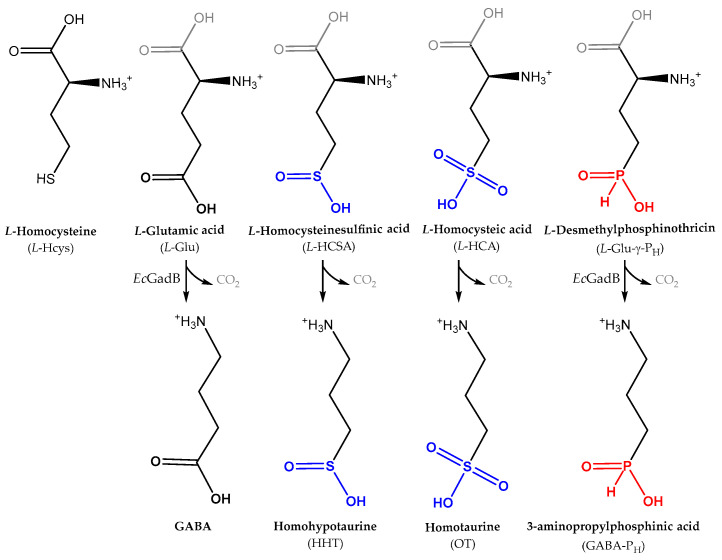
Chemical structures of *l*-homocysteinsulfinic acid (*l*-HCSA) and homohypotaurine (HHT). The above compounds are analogs of *l*-glutamic acid (*l*-Glu) and GABA, respectively, which are shown on the left-hand side, and partially oxidized if compared with *l*-homocysteic acid and homotaurine, respectively, which are shown on the right-hand side. The *H*-phosphinic compounds *l*-desmethylphosphinothricin (*l*-Glu-γ-P_H_), an analog of *l*-Glu, and 3-aminopropylphosphinic acid (GABA-γ-P_H_), an analog of GABA, both previously shown to be the substrate and product of *Ec*GadB, are shown on the rightmost side. The different colors allow to distinguish the S-containing groups (blue) and the P-containing group (red) that replace the carboxyl group in distal position from C-alpha. The alpha carboxyl group that is removed by decarboxylation is shown in gray.

It is biochemistry textbook knowledge that the essential amino acid *l*-methionine (Met) is the precursor of the amino acid *l*-cysteine (Cys). Both amino acids contain a sulfur atom and are of key importance. Indeed, ATG (AUG in mRNA) is the start codon in the genes of all living organisms, meaning that it is the codon recognized by the ribosome to initiate protein synthesis with a Met (or N-formylMet, fMet, in bacteria and bacteria-derived organelles) in the N-terminal position. Conversely, Cys contains a reactive thiol group in its side chain, which explains its prominent role in some enzymatic catalytic mechanisms and in stabilizing proteins’ tertiary and quaternary structures as pairs of cysteinyl residues form disulfide bonds (also known as disulfide bridges) [1]. Notably, when *l*-methionine is condensed to adenosine (starting from an ATP molecule) by the enzyme methionine adenosyl transferase, S-adenosylmethionine (SAM) is formed. SAM is a well-known coenzyme of methyltransferases, i.e., enzymes that transfer the methionine methyl group to different acceptors such as nucleic acids, proteins, and lipids, and as part of the synthesis of molecules such as adrenaline, choline, creatine, and carnitine [2]. Following methyl transfer, SAM becomes S-adenosyl homocysteine, which is subsequently hydrolyzed to adenosine and *l*-homocysteine (*l*-Hcys; Figure 1) by the enzyme adenosyl homocysteinase. An important reaction carried out by the enzyme methionine synthase restores the methyl group provided by N^5^-methyltetrahydrofolate (THF) to vitamin B_12_ and reforms *l*-Met. To form N^5^-methyl-THF, NADPH is used by N^5^,N^10^-methyleneTHF reductase [3].

The above-described pathway is the major pathway for *l*-Hcys as it allows for the reformation of *l*-Met, which is an essential amino acid. However, *l*-Hcys metabolism can also take other paths, such as the cyclization to *l*-homocysteine thiolactone, the homocysteinylation of proteins (on thiol or amino groups), and the trans-sulfuration pathway to generate *l*-Cys, via the sequential activity of the enzymes cystathionine β-synthase and cystathionine γ-lyase. In addition to protein synthesis, *l*-Cys is required for the synthesis of key molecules such as major intracellular antioxidants, i.e., the tripeptide glutathione, the biologically irreplaceable vasodilator gasotransmitter H_2_S, and taurine. Notably, taurine is derived from the oxidation of the Cys thiol group, resulting in *l*-cysteic acid (*l*-CA), which then undergoes decarboxylation on its C-alpha. Alternatively, taurine can be formed through the partial oxidation of *l*-Cys to *l*-cysteinsulfinic sulfinic (*l*-CSA), followed by its decarboxylation on its C-alpha, yielding hypotaurine (HT), which is then fully oxidized to taurine. This particular topic has been addressed in several excellent reviews and book series, some of which are listed herein [2,4,5,6].

Similar to the thiol group of *l*-Cys, the thiol group of *l*-Hcys can undergo partial oxidation to produce *l*-homocysteinesulfinic acid (*l*-HCSA) or full oxidation to *l*-homocysteic acid (*l*-HCA) via metabolic pathways not as fully clarified as those for *l*-CSA and *l*-CA [4]. Notably, both molecules, i.e., *l*-HCSA and *l*-HCA, are analogs of *l*-glutamate (Figure 1). The results of recent studies have shown that *l*-HCSA increases in the serum metabolome of acute myocardial infarction patients [7] and in the serum metabolome of high-risk stroke patients [8]; in comparison, *l*-HCA levels were found to decrease in the saliva of patients with oral leukoplakia and oral squamous cell carcinoma [9]. The results of the above studies indicate that the oxidation of *l*-Hcys to *l*-HCSA and *l*-HCA is metabolically relevant in physiopathological conditions other than the established endogenous activity of these molecules in the mammalian CNS [10].

The decarboxylation products of *l*-HCSA and *l*-HCA, i.e., homohypotaurine (HHT) and homotaurine (OT), respectively (Figure 1), display a structural similarity with the major inhibitory neurotransmitter of the CNS, i.e., GABA. OT (or tramiprosate) is not reported in vertebrates but is naturally found in seaweed. OT has recently attracted significant interest because of its ability to pass the blood–brain barrier and act as a neuroprotector in mild cognitive impairment patients by not only inhibiting the formation of Aβ42 oligomers in Alzheimer’s disease patients [11] but also by inducing changes in cortical GABA transmission via GABA receptor-mediated mechanisms [12,13].

Compared to the wealth of literature on some of the above metabolites, to the best of our knowledge, HHT metabolism and biological activity in mammalian cells have been scarcely investigated and the literature on this topic is lacking [14]. Furthermore, HHT is not readily available from commercial suppliers, or, if available, it is very expensive. Its chemical synthesis involves the use of homocystamine as a starting reagent [15,16]. In this work, in line with the trend of exploiting more widely bio-based syntheses to pave the way for greener synthetic approaches, it is demonstrated how HHT can be effectively synthesized starting from *l*-HCSA, as shown by the authors of previous studies [17]. Unlike previous studies, highly pure recombinant *Escherichia coli* glutamate decarboxylase (*Ec*GadB; EC 4.1.1.15) was used in the present study. *Ec*GadB has been extensively characterized at the structural and biochemical levels [18,19]. Glutamate decarboxylase is one of the structural components of the major acid resistance system in *E. coli*; however, it is also found in many other bacteria [20,21,22]. *Ec*GadB displays maximal activity at a pH of 3.8–5.4, in the presence of chloride ions, and rapidly auto-inactivates above pH 5.7 because the C-terminal tail locks the active site by covalently binding the Lys275-PLP Schiff base [23,24]. *Ec*GadB has already been demonstrated to be effective either immobilized on alginate beads to synthesize GABA, starting from *l*-Glu, its physiological substrate (*l*-Glu in Figure 1) [25], or in solution for the enantioselective decarboxylation of *l*-desmethylphosphinothricin in a racemic mixture [26] (*l*-Glu-γ-P_H_ in Figure 1). The latter compound is an analog of glutamate with a *H*-phosphinic group (-PO_2_H_2_) replacing the γ-carboxyl group of glutamate. HHT, following its enzyme-based synthesis, was purified via ion exchange chromatography and tested on endothelial cells and cardiomyoblsts in vitro, on which it proved to be protective against oxidative stress. Furthermore, this work supports the notion that the sulfinic group, similar to the *H*-phosphinic group [26], is a bioisostere of the carboxyl group as evidenced by the fact that, in addition to *Ec*GadB, other enzymes that recognize distal carboxyl groups, such as those in GABase, are able to metabolize compounds containing a sulfinic group.

## 2. Results

### 2.1. l-HCSA Is a Substrate of EcGadB and HHT Is a Substrate of GABase

*Ec*GadB has a specific activity of approx. 200 U/mg with its physiological substrate, the amino acid *l*-glutamate (Table 1). The enzyme is neither active on *d*-glutamate nor inhibited by its product, i.e., GABA [25,27,28]. The only natural substrates that in the 1970s were reported to be decarboxylated by bacterial glutamate decarboxylase are l-HCSA and *l*-HCA (Figure 1), though both to a smaller extent compared with *l*-Glu [29]. This convincing past evidence, combined with the strong and more recent evidence that the *H*-phosphinic group of *l*-Glu-γ-P_H_ (Figure 1) is a bioisostere of the distal carboxyl group of *l*-Glu for *Ec*GadB and for GABase (a commercial preparation of GABA-transaminase and succinic semialdehyde dehydrogenase) [26], provides the basis for a detailed analysis of the kinetic parameters of *Ec*GadB in the presence of *l*-HCSA.

**Figure 2:**
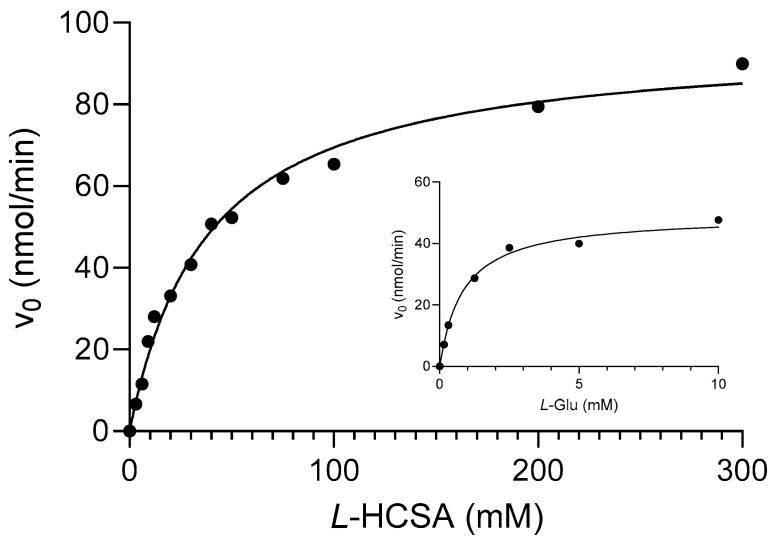
Rate of NADPH formation using the GABase assay on *l*-HCSA and *l*-Glu (inset) decarboxylation products using GABase. The rate corresponds to the HHT (or GABA for inset) produced during the assay at 37 °C in 0.1 M pyridine/HCl buffer, pH 4.6, containing 1 mM PLP and 0.1 mM DTT (see Section 4.3). The concentrations of *l*-HCSA used were in the range of 3 mM to 300 mM. The nmoles of product/min were obtained using 2.5 µg (0.5 µg for *l*-Glu) of *Ec*GadB in a final volume reaction mix of 100 µL (250 µL for *l*-Glu). The results are the average of two independent experiments. *l*-Glu is the *Ec*GadB physiological substrate. In both series of experiments, the standard deviation ranged from 0.7 to 6.2% of the experimental value shown on the graph.

In the present study, *l*-HCSA was synthesized and purified as described in Section 4 following a well-established protocol that yielded 306.5 mg of crystalline *l*-HCSA [16,30]. To monitor the HHT produced during the decarboxylation reaction on the C-alpha of *l*-HCSA by recombinant *Ec*GadB, NADPH formation was determined at each indicated time point by measuring the 340 nm absorbance following the GABase assay. The assay quantitatively yields NADPH due to HHT transamination and irreversible oxidation to 3-sulfinopropionic acid via GABase (see Section 4.3). For analytical purposes, the purity of *l*-HCSA was assessed and a preliminary analysis of HHT formation in the presence of *Ec*GadB was also performed via thin-layer chromatography (i.e., TLC, see Figure S1). To avoid overloading the thin layer, only aliquots of *l*-HCSA from 1 to 50 mM were spotted. The rate product formation at each *l*-HCSA concentration tested and the fitting of the data using the Michaelis–Menten equation are provided in Figure 2, together with the similar experiment performed on *l*-Glu, the *Ec*GadB physiological substrate (Figure 2, inset), for comparative purposes.

The results of the fitting analysis are reported in Table 1, where the kinetic parameters for *l*-HCSA are compared with those of *l*-Glu (Figure 2, inset) and *l*-Glu-γ-P_H_. [26].

The kinetic parameters indicate that the catalytic efficiency of *Ec*GadB in decarboxylating *l*-HCSA is 116 times lower than that on the physiological substrate (*l*-Glu) and 2.5 times greater than that on the phosphinic analog *l*-Glu-γ-P_H_. This difference is primarily due to an improvement in *k*_cat_, given that the *K*_M_ for the two analogs of *l*-Glu is almost identical.

### 2.2. Bioconversion of l-HCSA into HHT via EcGadB and HHT Purification

**Figure 3:**
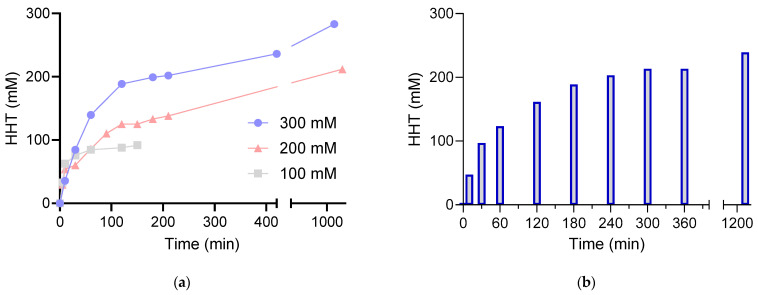
Bioconversion of *l*-HCSA into HHT by *Ec*GadB. The reaction was carried out at 37 °C in 0.2 M pyridine/HCl buffer, pH 4.6, containing 1 mM PLP and 1 mM DTT, and the pH was controlled via manual addition of a few microliters of 3 N HCl when necessary. *Ec*GadB was used at a concentration of 0.25 mg/mL. (**a**) Small-scale experiment (200 µL): HHT formation (mM) was measured at different time intervals using GABase over a duration of 18–19 h. (**b**) Preparative experiment (2.5 mL): HHT formation was measured at different time intervals using GABase over a time of 22 h (1320 min). At each of the indicated time points, an aliquot (5 µL) of the reaction was withdrawn and stopped via transfer into 495 µL of 100 mM HEPPS, pH 8.6, and then assayed with GABase (**a**,**b**).

Having established that HHT can be reliably quantitated using GABase (see Section 4.3 and Section 2.1 above), the next step consisted of establishing and fine-tuning the conditions required to produce HHT with high yields, with the aim of purifying this compound at the end of the bio-based synthetic process. Figure 3a shows a graph representative of small-scale (200 µL) bioconversion experiments at different concentrations of *l*-HCSA. 

Regardless of the starting concentration of *l*-HCSA used, i.e., 100 mM, 200 mM, and 300 mM, almost complete conversion (92–96%) into HHT was achieved. However, when *l*-HCSA was used at a starting concentration of 200 mM or 300 mM, it was necessary to allow the reaction to continue overnight to achieve maximal conversion (Figure 3a).

Given the high starting concentration of *l*-HCSA (i.e., 300 mM) and the correspondingly high proton consumption during its decarboxylation to yield HHT, it was necessary to check the pH and correct it via manual additions of small volumes of HCl (3N). Indeed, the presence of 0.2 M pyridine/HCl buffer, pH 4.6, was not sufficient to maintain the pH while the reaction proceeded over time. However, it was necessary to maintain the pH at 4.6–5.0 to avoid the widely recognized spontaneous inactivation of *Ec*GadB occurring at a pH above 6 [23,24,26].

The results shown in Figure 3a provided the basis for setting up a reaction for a preparative scale (i.e., 2.5 mL, 12.5 times the volume used for the small-scale experiments in Figure 3a) using 300 mM of *l*-HCSA (i.e., 125.3 mg in 2.5 mL). The formation of HHT was monitored over time by withdrawing aliquots of the reaction mixture at each indicated time point and assessing the HHT concentration with the GABase assay. The results shown in Figure 3b confirm that HHT formation occurred; nevertheless, by increasing the reaction volume, the final HHT yield resulted in being lower than in the small-scale setup and reached 80%. The analytical thin-layer chromatography results, where more time points were checked, confirmed that the conversion was indeed not complete (Supplementary Figure S2). Given that the primary objective of the above experiments was to obtain pure HHT to perform in vitro experiments, the 80% conversion yield was considered satisfactory to proceed with the next step, i.e., the purification of HHT via ion exchange chromatography. Therefore, after 22 h, the reaction was stopped by passing the reaction mixture through an ultrafiltration device, which removed the enzyme from the solution; thereafter, the ultrafiltrate was subjected to ion exchange chromatography on Dowex 50 X8, H^+^, previously described to be effective in purifying HHT [15,16]. The entire procedure yielded 50 mg of HHT, which corresponded to a 40% yield. The mass spectrum of the purified HHT confirmed its mass, i.e., calculated mass of 123.96 Da vs. expected monoisotopic mass of 123.03 Da (Supplementary Figure S3). Furthermore, HHT was assayed with the GABase assay, which confirmed the expected value when using a solution at a given concentration.

### 2.3. In Vitro Effect of HHT on Endothelial Cells and Cardiomyoblasts

**Figure 4:**
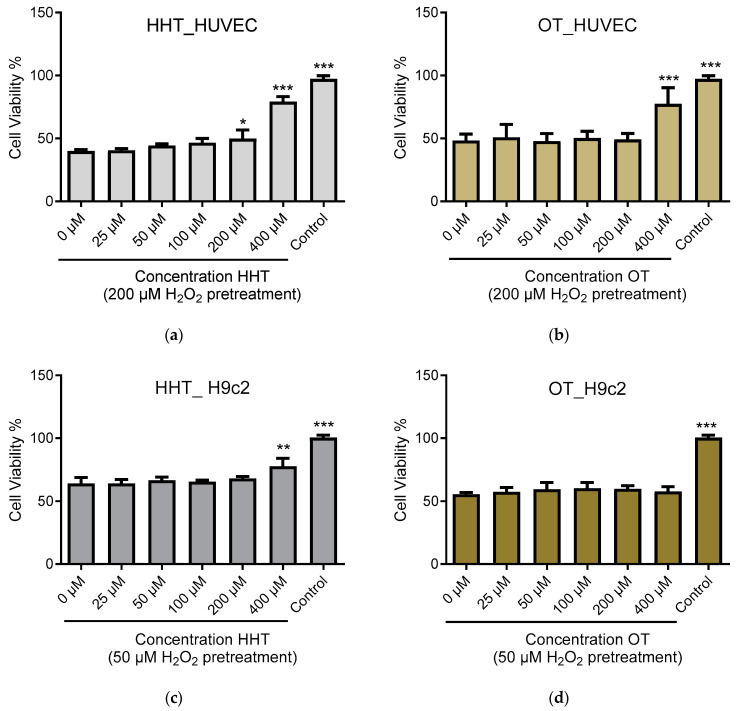
Effects of 24 h of treatment of HHT and OT on the viability of HUVEC and H9c2 cells pretreated with H_2_O_2_ for 4 h. Cell viability was assessed via the MTT assay. The treatment concentrations of HHT and OT are shown in the range of 0 to 400 µM. Oxidative stress was applied by pretreating HUVEC with 200 µM H_2_O_2_ and H9c2 with 50 µM H_2_O_2_ for 4 h. Data are displayed as the mean ± standard deviation from a minimum of 3 and a maximum of 6 independent experiments and indicate the % of viability over the untreated (0 µM) group. (**a**) cell viability, in the presence of the indicated concentrations of HHT, of HUVEC pretreated with 200 µM H_2_O_2_ for 4 h, (**b**) cell viability, in the presence of the indicated concentrations of OT, of HUVEC pretreated with 200 µM H_2_O_2_ for 4 h, (**c**) cell viability, in the presence of the indicated concentrations of HHT, of H9c2 pretreated with 50 µM H_2_O_2_ for 4 h, (**d**) cell viability, in the presence of the indicated concentrations of OT, of H9c2 pretreated with 50 µM H_2_O_2_ for 4 h. For the statistical analysis, one-way ANOVA followed by Tukey’s test was used. The statistical significance is given as * *p* < 0.05, ** *p* < 0.01, and *** *p* < 0.001. The control condition refers to the cells not challenged with H_2_O_2_ and is used herein as a reference for full viability.

HHT and OT have never been reported to occur in mammalian tissues; however, the presence of their precursors, i.e., *l*-HCSA and *l*-HCA, respectively, has been described in rat brains and also previously proposed by other authors [31,32]. Furthermore, the present work suggests that HHT and OT could be produced by the human microbiota through microbial enzymes such as *Ec*GadB, given that glutamate decarboxylase is an enzyme widely found in enteric bacteria [19,21]. Moreover, the structural similarity of HHT and OT with GABA (Figure 1), the reported binding and activity of HHT and OT to GABA receptors [13,33], and the evidence that GABA controls endothelial signaling and is protective against ROS [34,35] prompted our investigation into the biological activity of HHT and OT. Figure 4 shows the effects of both molecules in in vitro experiments involving human umbilical vein endothelial cells (HUVECs) and rat cardiomyoblasts (H9c2) pre-exposed to H_2_O_2_ to induce oxidative stress.

In the HUVEC cells (Figure 4a), following H_2_O_2_ pretreatment, cell viability was significantly higher when the cells were exposed to 200 µM (*p* < 0.05) and 400 µM of HHT (*p* < 0.001). When the same experiment was conducted with OT (Figure 4b), the positive effect was only observed in the presence of 400 µM of OT (*p* < 0.001). When rat cardiomyoblasts (H9c2) were pre-exposed to H_2_O_2_ and their viability was measured in the presence of increasing concentrations of both compounds, only HHT (Figure 4c) had a significant effect at 400 µM (*p* < 0.01); in comparison, treatment with OT did not show a significant effect (Figure 4d) among the studied concentration groups (*p* > 0.5).

## 3. Discussion

The present work provides evidence that pure recombinant *Ec*GadB could be an excellent tool for the bio-based synthesis of HHT, as already evidenced for GABA and its *H*-phosphinic analog [25,26]. The results of previous studies support this ability, though such studies were carried out with crude acetone powder of glutamate decarboxylase from *Clostridium welchii* ATCC13.124 and *E. coli* ATCC11246 [17,29]. The latter was subsequently demonstrated to be a mixture of two isoforms that were isolated and expressed separately from the relevant coding genes [36]. The availability of the pure recombinant *Ec*GadB isoform, which has been extensively studied at the biochemical and structural level, has made it possible to reconsider the results of previous studies with better knowledge of the structure–function relationship of the enzyme [18,19,23,24,26].

Notably, the available literature on HHT is very limited, and the possible metabolism (even in non-mammals) and biological effects of HHT have not received significant attention [14] despite the fact that HHT is an analog of GABA and the higher homolog of hypotaurine (HT).

Thus, while the metabolism of HT and taurine has been intensively studied in mammals and this topic is receiving significant attention, with new enzyme activities being discovered [37,38] and new roles assigned/suggested for both HT and taurine [39,40], the same advancements do not apply to HHT. The most recent article on HHT dates back to 1997 when Chebib et al. showed that HHT was a potent partial agonist (K_D_ = 4.59 µM) of the GABA_C_ receptor [41]. As detailed in the Introduction (Section 1), the abundance of *l*-HCSA, which originates from the partial oxidation of *l*-Hcys as part of an alternative and less prevalent pathway of *l*-Hcys metabolism, may explain the difference in the prevalence of HT vs. HHT [14]. However, the structural similarity of HHT with GABA (Figure 1) and the growing interest in OT (tramiprosate) in neuroprotection in Alzheimer’s disease patients [11,12,13,42] raises many still-unanswered questions on the possible roles and biological activity that HHT may have. The findings of a recent report suggest that glutamate decarboxylase in the human gut microbiota can indeed be a source for this molecule, providing a basis for probiotic interventions as well [43].

Herein, we first demonstrate that the enzyme-based synthesis of HHT is technically feasible and can be further optimized by improving the pH control of the reaction with the aid of a pH-stat, which could more effectively replace operator-based pH control, as used in the present study. Optimization of the bio-based synthesis of HHT with a reduction in the time required to reach completion is indeed highly desirable. It should in fact be reiterated that HHT can undergo oxidation to OT, and this fact should be taken into account while purifying and working with this analog [17]. In a previous study, *Ec*GadB was shown to be effectively immobilized on alginate beads [25], and this finding suggests that a similar approach for establishing the larger-scale synthesis of HHT is indeed possible. Furthermore, although not the primary objective of the study presented herein, the possibility of quantitatively measuring HHT via the GABase assay also allowed us to demonstrate that the sulfinic group (-SO_2_H) is a bioisostere of the carboxyl group, similar to the *H*-phosphinic group [26]. In fact, the two enzymes in GABase include GABA-transaminase, a PLP-dependent enzyme as *Ec*GadB, and succinic semialdehyde dehydrogenase, an NADP-dependent enzyme. The above findings are interesting, given that, in addition to NADPH (detected during the GABase assay), the reaction also leads to the formation of 3-sulfinopropionic acid (see Materials and Methods 4.3 and [44]).

An interesting and novel finding reported in the present work is the significant recovery of viability achieved in vitro with HHT on both endothelial cells (HUVECs) and cardiomyoblasts (H9c2) following oxidative stress. Moreover, HHT was found to be more effective than OT. This activity was initially hypothesized based on previous work on HHT and OT binding to GABA receptors [13,33] and based on the evidence that GABA controls endothelial signaling and provides protection against ROS [34,35]. Thus, the findings reported herein are original and set the basis for more in-depth studies, aimed at identifying the molecular targets of HHT and the activated cascade responsible for the observed amelioration of viability following oxidative stress. Based on our findings, HHT restores the viability of endothelial cells and cardiomyoblasts in response to oxidative stress. In this respect, HHT may also promote faster recovery from myocardial infarction and stroke, as *l*-HCSA, its precursor, has been shown to act as a biomarker [7,8].

Given the paucity of literature on HHT, it is currently not possible to propose possible mechanisms or pathways; despite this limitation, an anti-oxidative stress response and a receptor binding mechanism can be hypothesized for HHT. Future work will shed light on this molecule as a potential drug.

## 4. Materials and Methods

### 4.1. Materials

The following reagents used in the present study were of the highest purity level (≥98%) and purchased from Merck-Sigma Aldrich (Merck Life Science S.r.l., Milan Italy): *l*-homocystine (*l*-Hcys), pyridoxal 5′-phosphate (PLP), *l*-glutamate (*l*-Glu), 3-amino-1-propanesulfonic acid (i.e., homotaurine, OT), and dithiothreitol (DTT). GABase was also acquired from Sigma-Aldrich (Merck Life Science S.r.l., Milan Italy). The Vivaspin (4 mL 30 kDa cutoff) ultrafiltration devices were purchased from Sartorius Stedim Lab Ltd., Stonehouse, UK. *Ec*GadB was prepared as previously described [36]. Recombinant *Ec*GadB was purified following a well-established protocol [24,36] and its specific activity was approx. 220 U/mg.

### 4.2. Preparation of l-HCSA and Purification of l-HCSA and HHT via Column Chromatography

*l*-HCSA was prepared following a well-established protocol [16,30]. Briefly, the chemical synthesis process began with *l*-homocystine, which was split and oxidized to *l*-HCSA in the presence of excess CuCl_2_ (copper chloride) at alkaline pH. Thereafter, the solution was neutralized via the addition of HCl, filtered, and evaporated to a concentration of 20 mL. Dowex 50 X8 chromatography (1 × 16 cm column, H^+^) was applied, and, following washing with water, *l*-HCSA was eluted with ammonium hydroxide and evaporated once again to 20 mL. Next, Dowex 1 chromatography (1 × 8 cm column, HCOO^−^) was employed, an elution of *l*-HCSA was obtained with HCl (following water passage), and lastly, the greasy residue (5 mL) was subjected to a second round of Dowex 50 chromatography (1 × 4 cm column, H^+^) and the eluted HCSA was then crystallized. The yield was 306.5 mg of *l*-HCSA starting from 1.34 g of *l*-homocystine.

Following the bio-based conversion of *l*-HCSA into HHT (see Section 4.4), the latter compound was purified via single-step ion exchange chromatography based on published protocols [15,16,17]. Briefly, the compound obtained by preparative bioconversion of *l*-HCSA dissolved in 1 M sodium hydroxide was loaded on a Dowex 50 X8 (1.5 × 18 cm column, H+). After washing with water, HHT was eluted with 1 M ammonium hydroxide. The alkaline fractions reactive in the potassium permanganate test were collected and evaporated. The oily residue was repeatedly dissolved in absolute ethanol and dried until a crystalline residue was obtained. LC-DAD-MS determination of HHT was performed on a UPLC system (Waters, Milford, MA, USA), including a quaternary solvent manager (QSM), a sample manager with a flow-through needle system (FTN), a photodiode array detector (PDA), and a single–quadruple mass detector with an electrospray ionization source (ACQUITY QDa). Chromatographic analysis was performed on a C18 analytical column (Phenomenex). Solvent A was 0.1% aqueous HCOOH and solvent B was 0.1% HCOOH in CH_3_CN. The flow rate was 0.5 mL/min, and the elution was performed isocratically with 2% B for the first minute; thereafter, a gradient was applied. A DAD detector was set up in the range of 200 to 600 nm. Mass spectrometric detection was set in positive electrospray ionization mode using nitrogen as the nebulizer gas. Analyses were performed in Total Ion Current (TIC) mode in a mass range of 105 to 500 m/z. The capillary voltage was 0.8 kV, the cone voltage was 15 V, the ion source temperature was 120 °C, and the probe temperature was 600 °C.

### 4.3. Enzymatic Assays and Calculation of Kinetic Parameters

**Scheme 1:**
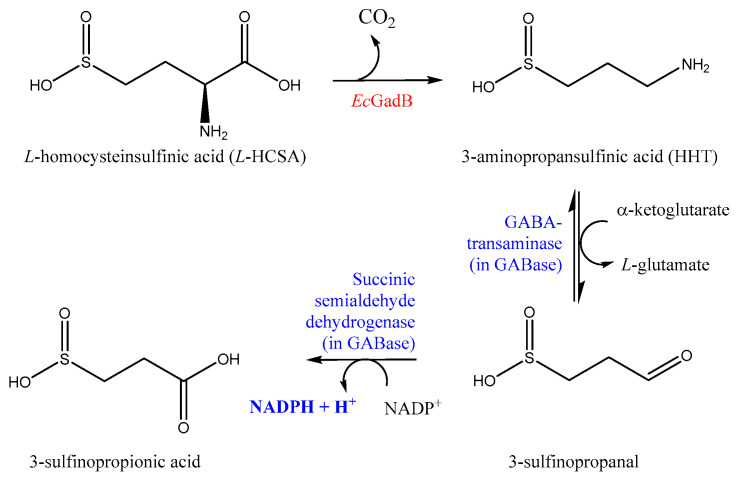
Sequence of the three uncoupled enzymatic reactions. The first irreversible reaction is carried out by *Ec*GadB (in red), at pH 4.6, on *l*-HCSA and yields HHT. The other two reactions are carried out by enzymes in GABase (in blue), of which the second is also irreversible and yields 3-sulfinilpropionic acid and NADPH (bold in blue), which can be detected spectrophotometrically (Abs_max_ = 340 nm).

To determine the best reaction conditions, the *k*_cat_ and *K*_M_ values for *l*-HCSA were determined in the buffer system of 0.2 M pyridine/HCl, pH 4.6, containing 1 mM of PLP and 0.1 mM of DTT. *Ec*GadB (2.5 μg) was incubated with *l*-HCSA (from 3 to 300 mM) in a final reaction volume of 100 μL. The rates of HHT formation were determined by quenching the reaction, which involved transferring aliquots (20 μL) of the reaction mixture into 100 μL of 0.1 M HEPPS buffer, which was pH 8.6, immediately followed by vigorous vortexing. At each tested concentration, three time points were determined (i.e., 2, 4, and 6 min). The HHT content was assayed with the GABase assay by transferring 20 μL of each halted reaction into 100 μL of GABase solution [36] containing 1.25 μL of activated GABase (0.02 units/μL). Incubation was carried out for 60 min at 37 °C to facilitate the transamination and quantitative oxidation by GABase of HHT into 3-sulfinopropionic acid and the concomitant formation of NADPH (Scheme 1). NADPH was quantified by measuring its 340 nm absorbance and applying the molar absorption coefficient (ε) NADPH_340_ = 6220 M^−1^ cm^−1^. The NADPH readings produced at each time point (i.e., 2, 4, and 6 min), by each *l*-HCSA concentration, were fitted by linear regression and converted back into nmoles of product/min. The calculated rates at each substrate concentration were then fitted to the standard Michaelis–Menten equation in GraphPad Prism 8 (GraphPad Software, San Diego, CA, USA).

A similar experimental setup was used to retrieve the same kinetic parameters for *l*-Glu for use in a comparative experiment. The only differences consisted in the amount of *Ec*GadB used (0.5 μg) and the range of concentration of *l*-Glu (from 0.15 to 10 mM, each tested at three time points 1, 2, and 3 min), in a final reaction volume of 250 μL. These changes were necessary to compensate for the much faster decarboxylation of *l*-Glu by *Ec*GadB (see Table 1).

### 4.4. Bioconversion of l-HCSA into HHT by EcGadB

The reaction mixture (200 μL for analytical purposes and 2.5 mL for preparative purposes) consisted of 0.2 M of pyridine/HCl buffer, pH 4.6, containing 1 mM of PLP, 1 mM of DTT, and 0.1, 0.2, and 0.3 M of *l*-HCSA, to which *Ec*GadB was added to a final concentration of 0.25 mg/mL. The analytical reactions (200 μL) were performed at 37 °C, and at the specified time intervals, aliquots were withdrawn for GABase analysis and/or thin-layer chromatography (TLC). During the reaction, the pH was periodically adjusted to 4.0–5.0 via stepwise addition of 3 N HCl (11–14 μL). At each given time point, 2 μL aliquots were transferred into 60 μL of 50 mM NaOH for TLC analysis, and 5 μL was transferred into 495 μL of 0.1 M HEPPS buffer, pH 8.6, immediately followed by vigorous vortexing, to measure the HHT content with the GABase assay (see Section 4.3). Given the fact that the aim of the small-scale experiments was to screen the best condition to run the preparative reaction, each procedure was performed once, except for the 0.3 M *l*-HCSA, which was run in duplicate for the first 3 h.

The preparative reaction (2.5 mL) was performed at 37 °C, adjusting the pH periodically to the value of 4.0–5.0 by adding 3 N HCl (total: 160 μL). At specified time intervals, aliquots (2 μL) were transferred into 60 μL of 50 mM of NaOH for TLC analysis (see Supplementary Figure S2), and 5 μL was transferred into 495 μL of 0.1 M HEPPS buffer, pH 8.6, immediately followed by vigorous vortexing to determine the HHT content with the GABase assay (see previous section). *Ec*GadB was separated via ultrafiltration on a Vivaspin (4 mL 30 kDa cut-off) ultrafiltration device.

### 4.5. Biological Activity of HHT and OT

#### 4.5.1. Cell Culture and Treatment Procedure

Human umbilical vein endothelial (HUVEC) and rat cardiomyoblastic (H9c2(2-1)) cell lines were acquired from the American Type Culture Collection (ATCC, USA). DMEM-F12 medium containing 10% fetal bovine serum and 1% penicillin–streptomycin (all from Gibco) was used for cell culture. Subculturing was performed with trypsinization every 2 to 3 days.

Pre-treatment of the cells (1 × 10^4^/well) with H_2_O_2_ was performed for 4 h to induce oxidative stress. The concentration of H_2_O_2_ was 200 µM for HUVEC and 50 µM for H9c2 cells, according to the previously determined IC_50_ of H_2_O_2_ (Supplementary Figure S4). Following removal of the medium containing H_2_O_2_, the cells were treated with HHT or OT dissolved in the culture medium for 24 h, where the studied concentrations were in the range of 0 to 400 µM. The control corresponded to the cells not challenged with H_2_O_2_ and let to grow in the culture medium; in comparison, the 0 µM concentration group corresponded to cells challenged with H_2_O_2_ for 4 h, and allowed to recover (if at all) without any treatment with HHT or OT.

#### 4.5.2. MTT Assay

After treatment, cell viability was measured with the 3-(4,5-Dimethylthiazol-2-yl)-2,5-Diphenyltetrazolium Bromide (MTT) assay [45]. A volume of 20 µL MTT solution (5 mg/mL in PBS, phosphate-buffered saline) was added to the cell medium and the cells were then incubated in the dark for 3 h. After removing the medium, formazan crystals were dissolved with 100 µL of DMSO. Absorbance was recorded at a wavelength of 590 nm.

#### 4.5.3. Statistical Analysis

Statistical analysis was performed with the program GraphPad Prism 6. Statistical differences in the treatment groups versus the untreated group (0 µM) were evaluated with one-way ANOVA followed by Tukey’s test. The results are given as the mean ± standard deviation (SD): * *p* < 0.05, ** *p* < 0.01, and *** *p* < 0.001.

## Figures and Tables

**Table 1:** Kinetic parameters of *l*-HCSA using *Ec*GadB.

Substrate	*k*_cat_ (s^−1^)	*K*_M_ (mM)	*k*_cat_/*K*_M_ (s^−1^ mM^−1^)
*l*-Glutamic acid (*l*-Glu)	86.78 ± 2.25	0.84 ± 0.09	103.31 ± 11.38
*l*-Homocysteinsulfinic acid (*l*-HCSA)	33.92 ± 1.07	38.24 ± 3.45	0.89 ± 0.09
*l*-Desmethylphoshinothricin (*l*-Glu-γ-P_H_) *	13.93 ± 0.58	39.82 ± 5.66	0.35 ± 0.05

* For comparison, the kinetic parameter of the *H*-phosphinic analog of *l*-Glu is provided [26].

## Data Availability

Access to relevant data can be requested from the authors upon reasonable and motivated request.

## Supplementary Materials

The following supporting information can be downloaded at https://www.mdpi.com/article/10.3390/molecules29173985/s1. Supplementary Figures S1–S4: Figure S1. *Time course of l-HCSA bioconversion into HHT by EcGadB*. The reactions were carried out using a range of concentrations of *l*-HCSA from 1 to 50 mM in 0.2 M pyridine/HCl buffer, pH 4.6, containing 1 mM PLP and 0.1 mM DTT. *Ec*GadB was used at a concentration of 0.06 mg/mL and each reaction was in a final volume of 110 µL. At each indicated time (including time 0, corresponding to no enzyme added), the reaction was halted by transferring 5 µL of the reaction mix into 10 µL of 50 mM NaOH and vortexed vigorously. 10 µL (for 0–1–3–5 mM condition) and 5 µL (for 10–15–20–40–50 mM condition) were spotted on thin layer silica sheet (on aluminium). Taking into account the dilution to halt the reactions, the minimum loading (time 0 at 1 mM *l*-HCSA) corresponded to 3.3 nmoles of *l*-HCSA whereas the maximum loading (time 0 at 50 mM *l*-HCSA) was 83.3 nmoles. On the leftmost part of the sheet, the blank (BK) with buffer only (0) and with the enzyme added (1) is shown as control, while on the rightmost part of the TLC 10 mM *l*-Glu (16 nmoles deposited) was reacted in the same conditions as above but only for 1 min, to yield GABA. The TLC was run in a vapor-saturated chamber, containing the solvent system isopropanol/ammonia 6.4 M/water in *v*/*v*/*v* ratio 7:0.32:2.68. The relative mobility of the compounds of interest are shown with arrows; the compounds (substrates and products) were detected by ninhydrin (0.2% in acetone) staining followed by drying with hot air to allow the development of the colored product. Figure S2. *Analytical TLC of the time course of the preparative scale bioconversion of l-HCSA into HHT by EcGadB*. The reaction was carried out starting from 300 mM *l*-HCSA which reacted for a total of 22 hours at 37 °C in 0.2 M pyridine/HCl buffer, pH 4.6, containing 1 mM PLP and 1 mM DTT, and controlling the pH via the addition, when necessary, of 3 N HCl (in total, 160 µl). *Ec*GadB was used at a concentration of 0.25 mg/mL in a final reaction volume of 2.5 mL. At each indicated time, the reaction was stopped by transferring 2 µL into 60 µL of 50 mM NaOH; 5 µL were spotted on thin layer sheet (silica on aluminium). The loading of *l*-HCSA at time 0 corresponded to approx. 40 nmoles, that of the control 5 mM *l*-HCSA corresponded to approx.0.8 nmole. TLC conditions and staining were as in the legend to Figure S1. Figure S3. *MS identification of purified HHT*. Chromatography was performed on a UPLC system as described in Materials and Methods (Section 4.2). Mass spectrometric detection was set in the positive electrospray ionization mode positive ESI [M-H]+, using nitrogen as nebulizer gas. Analyses were performed in Total Ion Current (TIC) mode in a mass range 105–500 m/z. Capillary voltage was 0.8 kV, cone voltage 15 V, ion source temperature 120 °C and probe temperature 600 °C. The mass spectrum of the purified HHT confirmed its mass, i.e. calculated mass 123.96 Da vs. expected monoisotopic mass 123.03 Da. Figure S4. Determination of IC_50_ of H_2_O_2_ on HUVEC and H9c2 cells. Two independent experiments were performed in triplicate for HUVEC cells (left graph); three independent experiments were performed in duplicate for H9c2(2-1) cells. The tested H_2_O_2_ concentrations were from 1 to 1000 µM. The IC_50_ calculated by non linear regression (curve fit of log[inhibitor] vs. normalized response—Variable slope), using GraphPad Prism were: 234.00 ± 2.30 µM for HUVEC cells and 44.75 ± 4.80 µM for H9c2 cells.
