# Supplementary material for: Synthesis and Biological Activity of Homohypotaurine Obtained by the Enzyme-Based Conversion of Homocysteine Sulfinic Acid Using Recombinant Escherichia Coli Glutamate Decarboxylase
Source: Molecules. 2024 Aug 23;29(17):3985. doi: 10.3390/molecules29173985 (PMC11396700; doi:10.3390/molecules29173985)
Supplement: Supplementary file 1 [file molecules-29-03985-s001.zip › molecules-3133185-supplementary.pdf]

molecules

Supplementary material

Synthesis and Biological Activity of Homohypotaurine Obtained by the Enzyme-Based Conversion of Homocysteine sulfinic acid Using Recombinant *Escherichia coli* Glutamate Decarboxylase

Mario Fontana [1], Aysenur Gunaydin Akyildiz [2], Chiara D'Alonzo [3], Fabio Giovannercole [3†], Arianna Zicchi [3‡], Antonio Francioso [1§], Elisabetta Capuozzo [1], and Daniela De Biase [3*].

[1] Department of Biochemical Sciences "A. Rossi Fanelli", Sapienza University of Rome, Piazzale Aldo Moro 5, 00185 Roma, Italy; mario.fontana@uniroma1.it (M.F.); elisabetta.capuozzo@uniroma1.it (E.C.); afrancioso@unite.it (A.F.).

[2] Department of Pharmaceutical Toxicology, Faculty of Pharmacy, Bezmialem Vakif University, 34093 Istanbul, Turkey; gunaydinaysenur@gmail.com (A.G.A.).

[3] Department of Medico-Surgical Sciences and Biotechnologies, Sapienza University of Rome, Corso della Repubblica 79, 04100 Latina, Italy; dalonzoc@gmail.com (C.D.); fabio.giovannercole@gmail.com (F.G.); ariannazicchi@gmail.com (A.Z.).

* Correspondence: daniela.debiase@uniroma1.it (D.D.B.).

† Present address: Biology of Microorganisms Research Unit (URBM), Namur Research Institute for Life Science (NARILIS), Université de Namur, Rue de Bruxelles 61, 5000 Namur, Belgium.

‡ Present address: Dipartimento di Scienze Mediche, Università di Torino, Corso Dogliotti 14, 10126 Torino, Italy.

§ Present address: Dipartimento di Bioscienze e Tecnologie Agro-Alimentari e Ambientali, Università degli Studi di Teramo, Campus di Coste Sant'Agostino, Via Renato Balzarini 1,Località Colleparco, 64100 Teramo, Italy.

Keywords: green chemistry; PLP; sulfinic compounds; HUVEC; H9c2; hydrogen peroxide.

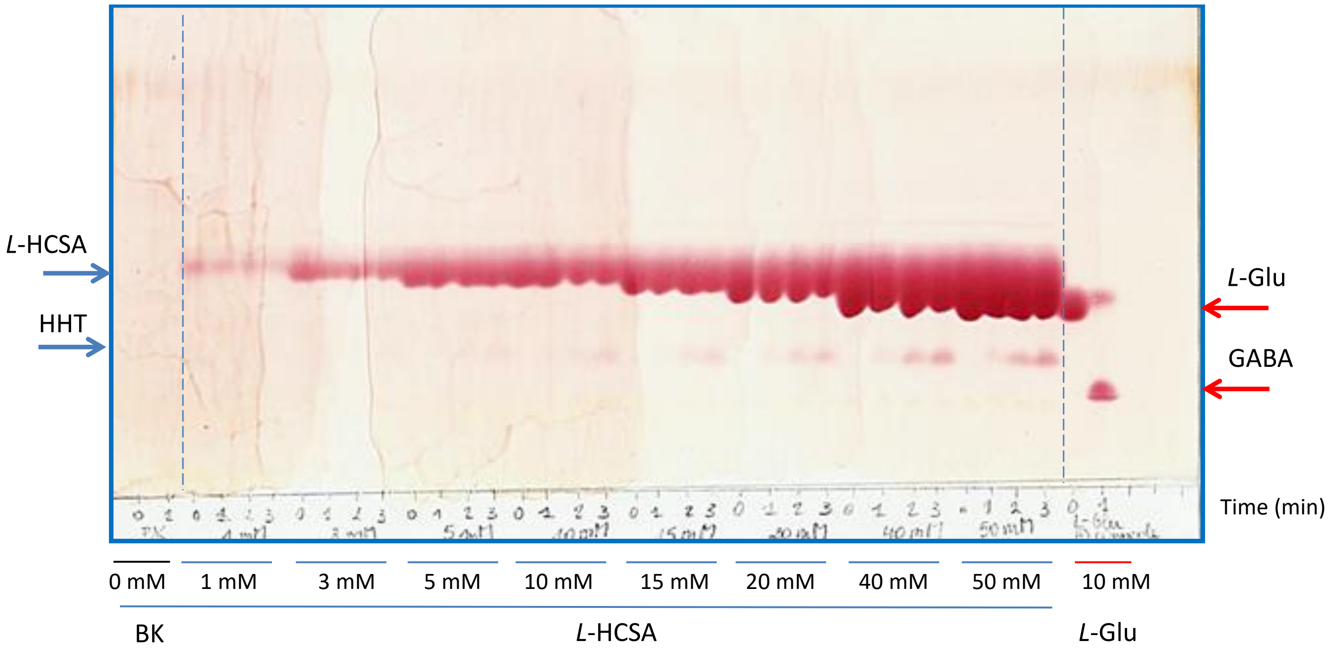

Figure S1. *Time course of L-HCSA bioconversion into HHT by EcGadB.* The reactions were carried out using a range of concentrations of *L*-HCSA from 1 to 50 mM in 0.2 M pyridine/HCl buffer, pH 4.6, containing 1 mM PLP and 0.1 mM DTT. *Ec*GadB was used at a concentration of 0.06 mg/mL and each reaction was in a final volume of 110 µl. At each indicated time (including time 0, corresponding to no enzyme added), the reaction was halted by transferring 5 µl of the reaction mix into 10 µl of 50 mM NaOH and vortexed vigorously. 10 µl (for 0-1-3-5 mM condition) and 5 µl (for 10-15-20-40-50 mM condition) were spotted on thin layer silica sheet (on aluminium). Taking into account the dilution to halt the reactions, the minimum loading (time 0 at 1 mM *L*-HCSA) corresponded to 3.3 nmoles of *L*-HCSA whereas the maximum loading (time 0 at 50 mM *L*-HCSA) was 83.3 nmoles. On the leftmost part of the sheet, the blank (BK) with buffer only (0) and with the enzyme added (1) is shown as control, while on the rightmost part of the TLC 10 mM *L*-Glu (16 nmoles deposited) was reacted in the same conditions as above but only for 1 min, to yield GABA. The TLC was run in a vapor-saturated chamber, containing the solvent system isopropanol/ammonia 6.4 M/water in v/v/v ratio 7:0.32:2.68. The relative mobility of the compounds of interest are shown with arrows; the compounds (substrates and products) were detected by ninhydrin (0.2% in acetone) staining followed by drying with hot air to allow the development of the colored product.

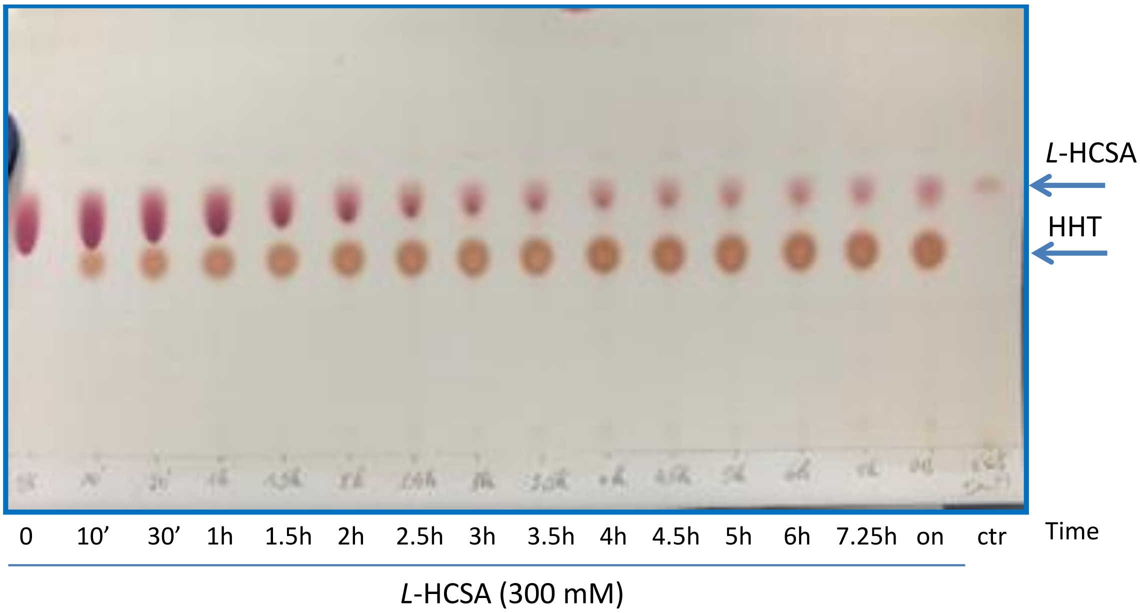

Figure S2. *Analytical TLC of the time course of the preparative scale bioconversion of L-HCSA into HHT by EcGadB*. The reaction was carried out starting from 300 mM *L*-HCSA which reacted for a total of 22 hours at 37°C in 0.2 M pyridine/HCl buffer, pH 4.6, containing 1 mM PLP and 1 mM DTT, and controlling the pH via the addition, when necessary, of 3 N HCl (in total, 160 µl). *Ec*GadB was used at a concentration of 0.25 mg/ml in a final reaction volume of 2.5 ml. At each indicated time, the reaction was stopped by transferring 2 µl into 60 µl of 50 mM NaOH; 5 µl were spotted on thin layer sheet (silica on aluminium). The loading of *L*-HCSA at time 0 corresponded to approx. 40 nmoles, that of the control 5 mM *L*-HCSA corresponded to approx.0.8 nmole. TLC conditions and staining were as in the legend to Figure S1.

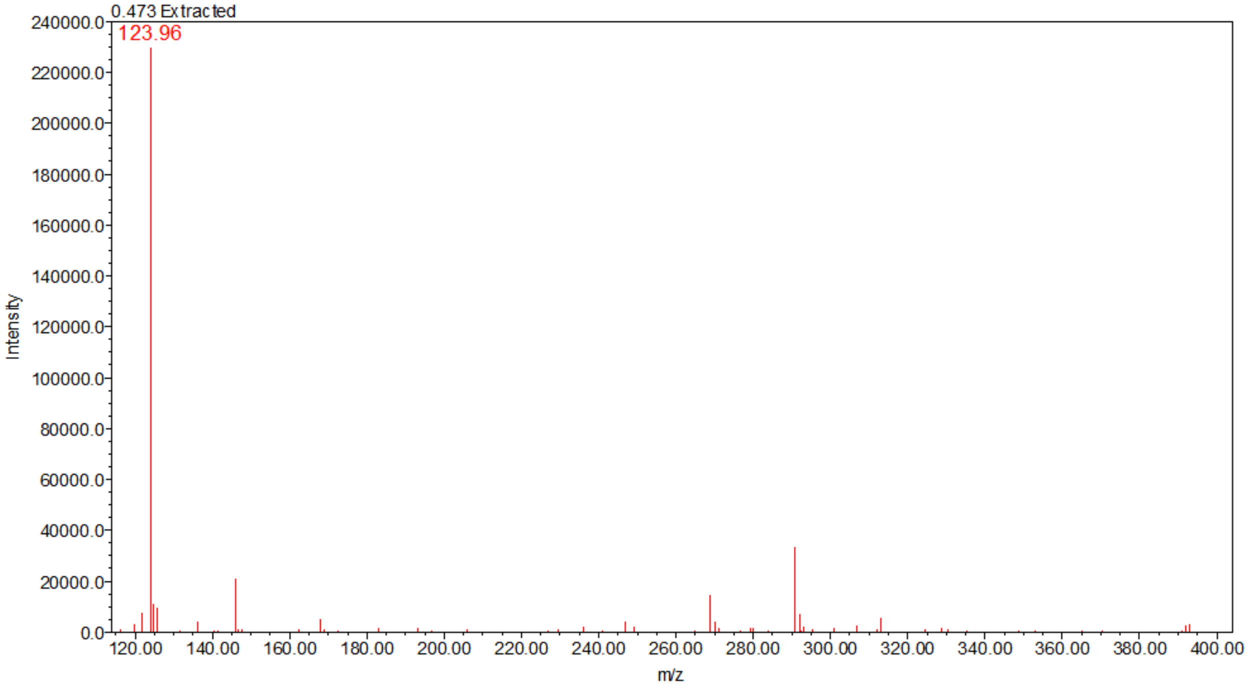

Figure S3. *MS identification of purified HHT*. Chromatography was performed on a UPLC system as described in Materials and Methods (subsection 4.2). Mass spectrometric detection was set in the positive electrospray ionization mode positive ESI [M-H]+, using nitrogen as nebulizer gas. Analyses were performed in Total Ion Current (TIC) mode in a mass range 105–500 m/z. Capillary voltage was 0.8 kV, cone voltage 15 V, ion source temperature 120 °C and probe temperature 600 °C. The mass spectrum of the purified HHT confirmed its mass, i.e. calculated mass 123.96 Da vs expected monoisotopic mass 123.03 Da.

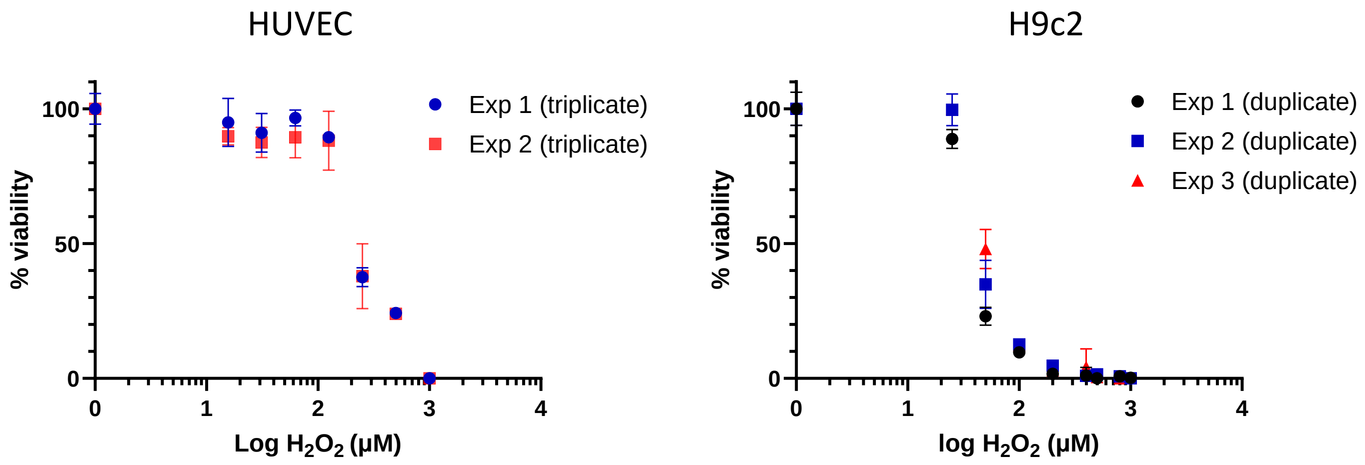

Figure S4. *Determination of IC_{50} of H_2O_2 on HUVEC and H9c2 cells*. Two independent experiments were performed in triplicate for HUVEC cells (left graph); three independent experiments were performed in duplicate for H9c2(2-1) cells. The tested H_2O_2 concentrations were from 1 to 1000 µM. The **IC_{50}** calculated by non linear regression (curve fit of log[inhibitor] vs. normalized response - Variable slope), using GraphPad Prism were: **234.00 ± 2.30 µM for HUVEC** cells and **44.75 ± 4.80 µM for H9c2** cells.
